# Co-design of an Electronic Dashboard to Support the Coproduction of Care in Pediatric Rheumatic Disease: Human-Centered Design and Usability Testing

**DOI:** 10.2196/34735

**Published:** 2022-04-22

**Authors:** Alysha Taxter, Lisa Johnson, Doreen Tabussi, Yukiko Kimura, Brittany Donaldson, Erica Lawson, Vincent Del Gaizo, Daniela Vitelli, Corinne Pinter, Aricca Van Citters, Eugene Nelson, Tzielan Lee

**Affiliations:** 1 Nationwide Children's Hospital Columbus, OH United States; 2 Dartmouth College Lebanon, NH United States; 3 Hackensack University Medical Center Hackensack, NJ United States; 4 Wake Forest University Winston-Salem, NC United States; 5 University of California San Francisco San Francisco, CA United States; 6 Childhood Arthritis and Rheumatology Research Alliance (CARRA) Milwaukee, WI United States; 7 Parent Partner Hasbrouck Heights, NJ United States; 8 Parent Partner Sugar Land, TX United States; 9 Stanford Children’s Health Palo Alto, CA United States

**Keywords:** human-centered design, coproduction, dashboard, pediatric rheumatology, juvenile idiopathic arthritis, JIA, juvenile arthritis, patient centered, patient-reported outcomes, patient communication, patient education, family education

## Abstract

**Background:**

The coproduction of care involves patients and families partnering with their clinicians and care teams, with the premise that each brings their own perspective, knowledge, and expertise, as well as their own values, goals, and preferences, to the partnership. Dashboards can display meaningful patient and clinical data to assess how a patient is doing and inform shared decision-making. Increasing communication between patients and care teams is particularly important for children with chronic conditions. Juvenile idiopathic arthritis (JIA), the most common chronic pediatric rheumatic condition, is associated with increased pain, decreased function, and decreased quality of life.

**Objective:**

The aim of this study is to design a dashboard prototype for use in coproducing care in patients with JIA. We evaluated the use and needs of end users, obtained a consensus on the necessary dashboard data elements, and constructed display prototypes to inform meaningful discussions for coproduction.

**Methods:**

A human-centered design approach involving parents, patients, clinicians, and care team members was used to develop a dashboard to support the coproduction of care in 4 ambulatory pediatric rheumatology clinics. We engaged a multidisciplinary team (n=18) of patients, parents, clinicians, nurses, and staff during an in-person kick-off meeting followed by biweekly meetings. We also leveraged advisory panels. Teams mapped workflows and patient journeys, created personas, and developed dashboard sketches. The final dashboard components were determined via Delphi consensus voting. Low-tech dashboard testing was completed during clinic visits, and visual display prototypes were iterated by using the Plan-Do-Study-Act methodology. Patients and clinicians were surveyed regarding their experiences.

**Results:**

Teams achieved consensus on what data mattered most at the point of care to support patients with JIA, families, and clinicians collaborating to make the best possible health care decisions. Notable themes included the right data in the right place at the right time, data in once for multiple purposes, patient and family self-management components, and the opportunity for education and increased transparency. A final set of 11 dashboard data elements was identified, including patient-reported outcomes, clinical data, and medications. Important design considerations featured the incorporation of real-time data, clearly labeled graphs, and vertical orientation to facilitate review and discussion. Prototype paper-testing with 36 patients and families yielded positive feedback, with 89% (8/9) to 100% (9/9) of parents (n=9) and 80% (8/10) to 90% (9/10) of clinicians (n=10) strongly agreeing or agreeing that the dashboard was useful during clinic discussions, helped to talk about what mattered most, and informed health care decision-making.

**Conclusions:**

We developed a dashboard prototype that displays patient-reported and clinical data over time, along with medications that can be used during a clinic visit to support meaningful conversations and shared decision-making among patients with JIA, their families, and their clinicians and care teams.

## Introduction

### Background

The coproduction of care involves patients and families partnering with their clinicians and care teams, with the premise that each brings their own perspective, knowledge, and expertise, as well as their own values, goals, and preferences, to the partnership. Inviting and integrating these unique strengths support effective patient-family-clinician relationships [1-3]. Recent studies have shown that these partnerships can also be aided by implementing dashboards that display meaningful data that can be reviewed together at the point of care to assess a patient’s progress and to make shared treatment decisions, particularly for patients and families living with chronic illnesses [4-6].

Juvenile idiopathic arthritis (JIA) is the most common chronic rheumatic condition, affecting 1 in 1000 [7,8] children. Even with advances in treatment options such as biological medications, children with JIA have decreased physical function, worse health-related quality of life, and increased pain despite improved disease activity [9-11]. Although the pediatric rheumatology field routinely collects patient-reported outcomes (PROs) of pain, function, and disease activity for research and collaborative improvement purposes [12,13], these data are not regularly integrated into clinical practice to inform care and treatment decisions. Growing evidence suggests that leveraging such data at the point of care can lead to improved health outcomes, which are of critical importance to children and families living with JIA. These families manage complex treatment regimens and regular visits with multiple subspecialists, including ophthalmology, psychology, and physical and occupational therapists, and usually require the long-term use of injectable and infusion therapies [14]. It is also known that children, parents, and clinicians have different views of disease and expectations of treatment outcomes, and it is important to enable children to actively communicate their views with their clinician [15].

Dashboard data visualization tools are used in health care to aggregate and integrate key data for review and discussion during clinical encounters to support patient-centered care. The Swedish Rheumatology Quality Register dashboard serves as a long-standing rheumatology model [16]. It integrates and displays PROs (eg, pain, global health, and fatigue), key clinical data (eg, joint count and disease activity scores), and treatments and medications longitudinally and has been used by patients and their clinicians since 2004 to engage in coproduction of care. The Swedish Rheumatology Quality approach was associated with a 50% decrease in disease activity between 2004 and 2014 in people living in Sweden with rheumatoid arthritis (RA) [17].

Dashboard use in the US rheumatology community is increasing, building on earlier work in the field to provide data for clinical decision support at the point of care [18]. Design efforts have been completed at the University of California, San Francisco, health system to support an electronic health record (EHR)-based patient-facing dashboard for adult patients with RA [19,20]. However, we are not aware of similar efforts in patients with pediatric rheumatology and their families. Given the dearth of evidence-based care protocols in pediatric rheumatology, these patients and their families face an even greater need to bring together PROs and key clinical data in one place to support shared decision-making. To address this gap, we developed a human-centered co-design process to create a prototype of an electronic JIA dashboard.

### Objective

Our objective was to design a real-time point-of-care dashboard to support partnerships between patients and families and their clinicians by identifying the data and information that matter most to them and designing the display for enhanced communication and decision-making.

## Methods

### Overview

**Figure 1:**
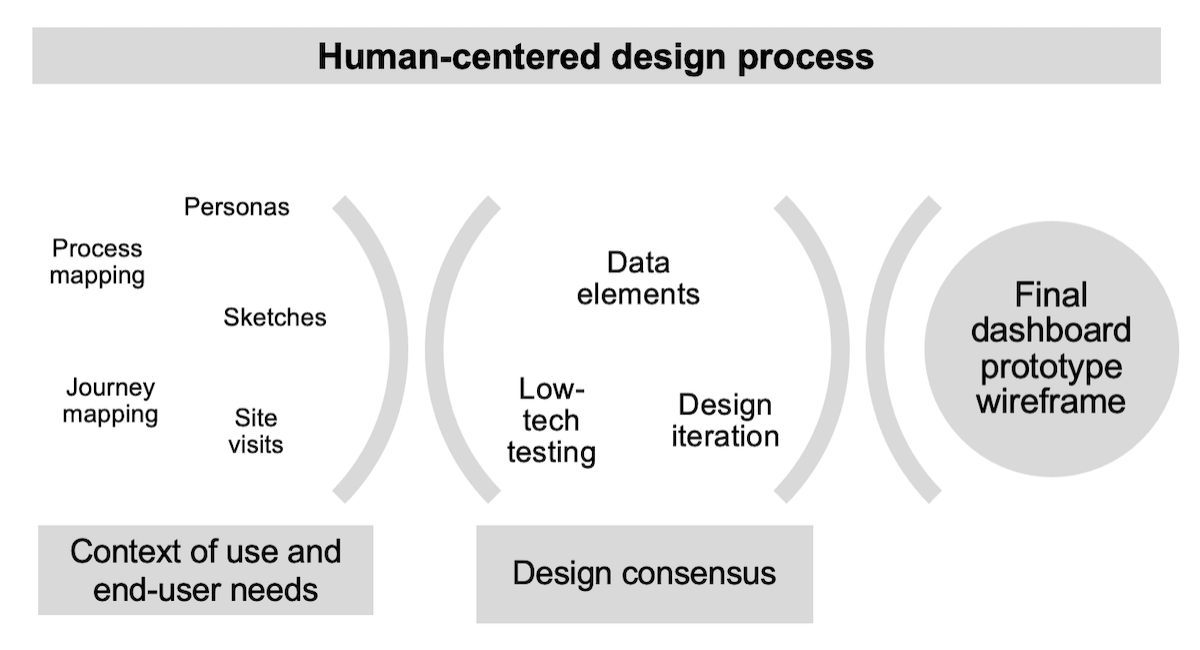
Human-centered design process.

Our study was guided by a human-centered design process [21] to ensure that the final dashboard design would serve the needs and goals of end users. The process involved deploying a series of iterative methods to (1) explore the context of use and needs of end users and (2) achieve consensus on the dashboard data elements and overall dashboard design (Figure 1).

### Ethics Approval

This study was approved by the Dartmouth College Institutional Review Board (#31341).

### Participants

The co-design process included clinical care team members (n=12: 3 physicians, 1 physician assistant, 2 advanced practice nurses, 2 registered nurses, and 4 other staff members), a teenager with JIA (n=1), and parents (n=5) from 3 US pediatric rheumatology sites (Hackensack University Medical Center, Stanford Children’s Health, and Wake Forest University). Sites were initially identified among members of the Childhood Arthritis and Rheumatology Research Alliance (CARRA) and the Pediatric Rheumatology Care and Outcomes Improvement Network (PR-COIN) organizations. Sites with strong clinical leadership and information-technology collaborations were chosen. All the sites had the same EHR vendor. A fourth US site (University of California, San Francisco) was added during the final dashboard design wireframe build.

Additional iterations on the dashboard design were guided by an 11-member parent partner advisory group that met monthly throughout the co-design phase. This group included 5 parent partners from the co-design sessions and 6 additional parent partners identified by the Arthritis Foundation. A clinical advisory group consisting of 5 additional clinicians provided further input in one 60-minute session.

The Dartmouth Institute for Health Policy and Clinical Practice led the facilitation and general leadership of this initiative.

### Exploring Contexts and End-User Needs

We held a 2-day meeting in March 2018 and convened leaders from the Arthritis Foundation, CARRA, PR-COIN, and 3 to 5 parent and clinical members from each of the pilot site teams. The meeting included working sessions for brainstorming, sketching a dashboard mock-up, and discussing desired dashboard uses and features from key stakeholder perspectives. Ensuring dashboard uptake by patients with JIA and their families, clinicians, and care teams served as a guiding tenet for our work.

Following the kick-off meeting, the pilot site teams met biweekly via videoconference for subsequent co-design sessions from April to December 2018. These sessions focused primarily on gaining an understanding of the context of dashboard use and the needs of end users, using human-centered design activities to ideate, explore, and observe. Activities included process mapping, generating personas, journey mapping, developing dashboard sketches, and visiting sites.

#### Process Mapping

To gain insight into their current state of care delivery and to visualize opportunities for coproduction between patients, families, and clinicians, each team created a flowchart [22] of their care processes and corresponding data flows for patients with JIA.

#### Personas

Each site developed three personas: a pediatric rheumatologist, a parent or family member, and a child or teenager with JIA. Personas are archetypes or examples of end users (in this case, the end users of the coproduction dashboard) and their patterns that can be used to inform and guide design decisions. They clarify the goals, behavior patterns, and needs of an end-user population and generate useful design targets [23]. Teams used a template to record persona elements, including interests, skills, goals, daily routines, likes and dislikes, motivation, context, and needs and desires. Teams were asked to translate multiple conversations and observations into a representative set of persona characteristics; however, some teams used *real life* individuals for their personas.

#### Journey Mapping

The parent partner advisory group participated in a journey mapping exercise [21] intended to capture the patient and family lived experience in the care journey. Journey mapping described “walking through a visit”—from preparing for the visit at home, arriving at the clinic, moving through the clinic visit, and checking out to following up afterward. Parent partners described actions, questions that needed answering, happy moments (things that improved the care experience), and pain points (frustrations and annoyances) for each step in the care process. The aim was to consider how to leverage happy moments and understand how to improve pain points in designing the dashboard innovation.

#### Dashboard Sketches

Teams engaged in a visual thinking exercise designed to generate ideas for dashboard design and invite commentary [24]. Each team member was instructed to draw a picture of their ideal dashboard, review and discuss it with their team, and compare and consolidate the best ideas to create a team dashboard. Similarities and differences across team sketches were noted and discussed during the co-design session.

#### Site Visits

The Dartmouth Institute for Health Policy and Clinical Practice team conducted a site visit at each pilot site to engage key stakeholders (team members, local leadership and informatics teams, and patients and families) in the dashboard design process and to observe clinical operations and care flows to better understand the context and workflow of the dashboard. They met with patients and families (n=12) in individual and group settings to provide an overview of the dashboard initiative and capture their feedback and ideas for enhancing the information environment at the point of care.

### Building Consensus

After exploring how a dashboard could support the needs and requirements of patients, families, clinicians, and care teams for use during clinic visits, teams engaged in determining the detailed design requirements for the dashboard. This process included finalizing the data elements, layout, and visual look and feel of the dashboard, and considering features to support self-management and other user needs.

#### Delphi Voting on Dashboard Data Elements

Clinical care team members, patients, and parents participated in a Delphi voting process via web-based surveys and multisite team meetings to achieve consensus on a parsimonious set of data elements to be displayed on the dashboard. An initial list of data elements and dashboard features was compiled based on personas, dashboard sketches, and discussions with patients, families, clinicians, and care teams during pilot site design sessions and site visits. Teams were also asked to review the list and suggest additional items that might be missing.

Dashboard elements were organized by domain in preparation for the 3 rounds of Delphi voting. The domains included PROs, clinical data, medications, self-management, and other user features. In the first 2 rounds, team members ranked the elements within each domain using a 5-point Likert scale. They also indicated their top 5 elements for aggregate reviews as a cross-check against the rankings. After the second round of voting, the domain containing self-management and other user features was removed from the final round of voting, as it was determined that the patient and family-facing self-management tool was outside the initial scope of the point-of-care dashboard.

In the third and final round of voting, team members were asked to prioritize the data elements based on modified MoSCoW (must have, should have, could have, will not have) criteria [25]: must have, nice to have, will not have (but nice someday), and not a priority. After the results from the third round of voting were tallied, a broader group of stakeholders—clinician advisory group, parent partner advisory group, and CARRA and PR-COIN registry leaders—were engaged in reviewing and offering feedback before the dashboard data elements were finalized.

Specific measures or tools for each PRO data element were reconciled and aligned with the data collection for the CARRA [12] and PR-COIN [26] registries, with the goal of data collected once and used for multiple purposes. In addition, measures or tools validated for use in pediatric rheumatology were identified as preferable.

#### Low-Tech Dashboard Testing and Design Iteration

Sites engaged in testing a paper-based version of the dashboard with a small number of clinical patients and families using the Plan-Do-Study-Act framework [27]. The aim of rapid-cycle testing was to assess the feasibility and utility of the dashboard at the point of care and to incorporate feedback to iteratively refine and enhance the dashboard design and usability within the flow of care.

The dashboard prototype was created using a Microsoft Excel [28] template to display patient-reported data and key clinical data obtained from patients completing previsit questionnaires and the EHR. It was introduced to patients with JIA and their families by a rheumatology clinician. Parents were surveyed after the visit to rate their overall impressions of the prototype dashboard. Clinicians were also surveyed regarding their experiences with the dashboard.

Following low-tech testing, a third-party digital health solution organization was engaged to translate the findings from the human-centered design process into a final wireframe of the dashboard design. Iterative design sessions were held to obtain feedback based on the preferences of the patients, families, clinicians, and care teams.

## Results

### Insights From Contexts and End-User Needs

Human-centered design activities demonstrated the needs of end users and the processes required to integrate a dashboard into the flow of clinical care. This iterative framework generated ideas and insights about the features and functionalities that are most important to the design of the dashboard. Several notable themes emerged from iterative discussions between the project facilitators and co-design teams throughout the co-design process: (1) the right data, in the right place, at the right time, (2) data in once for multiple purposes, (3) patient and family self-management components, and (4) opportunity for education and increased transparency.

#### The Right Data, in the Right Place, at the Right Time

The EHR serves as a cumulative repository of the data and information generated at each clinical encounter. Pilot site teams stressed they did not want to replicate the EHR and instead arrived at a balance of data to support the coproduction of care:

Making decisions about treatment plans and medications is complex and involves weighing pros/cons...need enough/sufficient information but not too overwhelming.

Clinician: multisite team meeting

**Figure 2:**
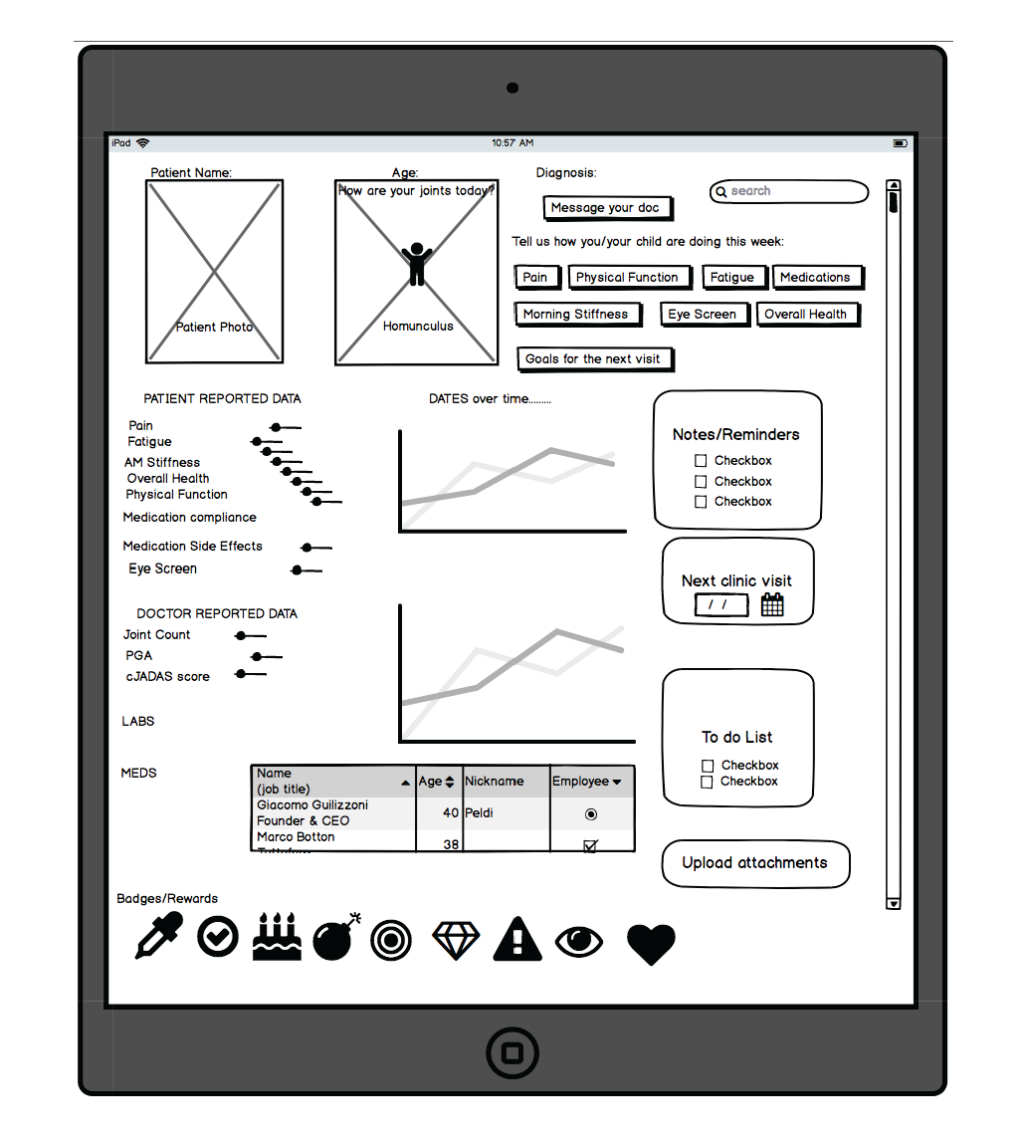
Example dashboard sketch. cJADAS: clinical juvenile arthritis disease activity score; PGA: physician global assessment.

Trending data over time (including medication usage) was identified as an essential functionality and included in all team dashboard sketches (Figure 2):

I would like to know how she is progressing better or worse over time.

Parent persona

In addition, the teams expressed a desire to personalize the dashboard with a patient photo and updates on life between visits:

We normally just see snapshots of patients in clinics; it would be great to have a bigger picture of what goes on in our patients’ lives on a day-to-day basis.

Clinician: multisite team meeting

#### Data in Once for Multiple Purposes

A frustration identified by patients and families during the journey mapping exercise was being asked the same questions repeatedly during a visit as well as completing questionnaires and then not seeing the results or understanding how the data were being used:

A goal is to have information that gets shared actually get to the provider—by the time we get to the clinician, only 10% of what we’ve shared at every step of the visit process actually gets to the doctor.

Parent: 2-day kick-off meeting

In addition, clinicians discovered that the collection of PROs typically occurred toward the end of the visit, with a research coordinator collecting the data for research registry purposes. Teams agreed that an important design specification would be to ensure that previsit questionnaire data both inform the clinic visit and populate registries.

#### Patient and Family Self-management Components

**Figure 3:**
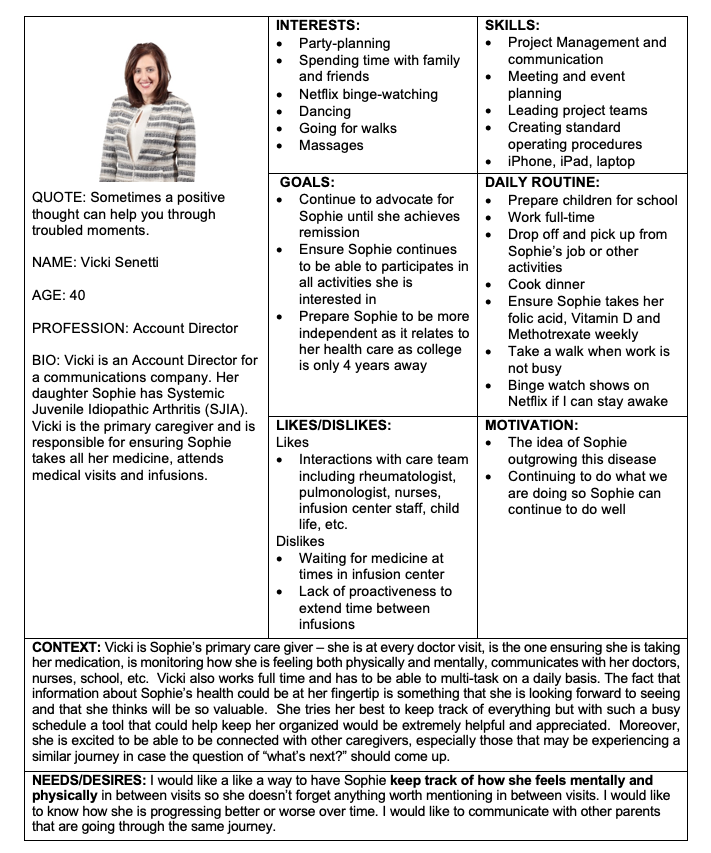
Parent persona.

Personas developed for this project, such as the example in Figure 3, provide insight into patient and family needs in managing chronic diseases. Families of children and teenagers with JIA desire a place to collect, track, and review disparate pieces of information and data needed to optimize the management of their child’s health:

I am looking forward to one place where my son’s health information is all in one place for me to see.

Parent persona

A space for children and teenagers with JIA to self-report on activities or symptoms important to them, a *to-do* list, and a medication tracker were cited as desirable self-management features:

I would like a way to keep track of how I feel mentally and physically in between visits so that I can let my doctor know, especially since my visits are spread out.

Patient persona

Furthermore, during the co-design sessions, parents shared the information that they routinely collected to prepare for a clinic visit, including laboratory work required and completed, questions and updates to share with the physician, and the date of their child’s last eye examination. They expressed the need to have this information centrally available for previsit planning.

#### Opportunity for Patient Education and Increased Transparency

Involving patients and parents alongside clinicians and care teams during the design process offered each group unique insight into the data and information most important to each in coproducing care. For example, parents were particularly concerned about medication side effects and lobbied to include a laboratory measure of liver toxicity (alanine aminotransferase) on the dashboard. Clinicians and care team members assured parents that they always reviewed alanine aminotransferase scores as part of every visit; however, they deferred to parent preference to include it on the dashboard. Similarly, patients and parents were unaware of measures used for clinical assessment, such as the Juvenile Arthritis Disease Activity Score (JADAS) [29]. Clinicians admitted that they did not typically explain the clinical and research importance of the JADAS, acknowledging that the dashboard would offer an opportunity for education and increased transparency with patients and families:

We would like to spend less time charting and dealing with insurance companies and more time with our patients on education and management of the disease, ensuring that we/they have a true understanding of their medical condition and treatments, daily life, and coping strategies. We want to promote self-reliance and self-management.

Clinician persona

### Consensus on Dashboard Design

#### Finalized Set of Data Elements

The Delphi method was used to reach consensus on the final set of dashboard data elements. The necessary data domains included PROs, key clinical data, and current and past medications. The initial round of voting started with 25 data elements: 13 in the PRO domain, 11 in the clinical data domain, and 1 associated with medications. After 3 rounds of voting, a consensus was reached on the 11 data elements summarized in Textbox 1.

Data elements that were initially considered, but did not achieve consensus, included fatigue, morning stiffness, mental health, uveitis status, and several laboratory values (C-reactive protein, erythrocyte sedimentation rate, and aspartate aminotransferase).

**Final dashboard data elements.**

**Patient-reported outcomes**

- Concerns, questions: free-text patient, parent, and family questions or concerns for discussion in the visit
- Patient global assessment: ordinal 0-10 scale, patient’s assessment of overall well-being
- Physical function: Patient Reported Outcomes Measurement Information System (PROMIS) v1.0 Pediatric Upper Extremity short form raw summed score; PROMIS v1.0 Pediatric Mobility short form raw summed score
- Pain, pain interference: ordinal 0-10 current rating of pain and PROMIS v1.0 Pain Interference Short Form raw summed score
- Medication adherence: 5-point Likert scale that indicates how often medications are being taken as prescribed and includes the option of “I am not currently taking any prescribed rheumatology medications.”
- Medication side effects: list of symptoms experienced with current medications

**Clinical data**

- Joint count: total number of tender joints and total number of swollen joints
- Provider global assessment: ordinal 0-10 scale (provider’s assessment of patient’s overall disease activity)
- Disease activity (Juvenile Arthritis Disease Activity Score): 0-30 composite score that incorporates patient global assessment, provider global assessment, and joint count
- Liver toxicity: alanine aminotransferase

**Medications**

- Medications: medication name, dose, route, start and stop dates, and frequency

#### Low-Tech Testing and Design Iteration

**Figure 4:**
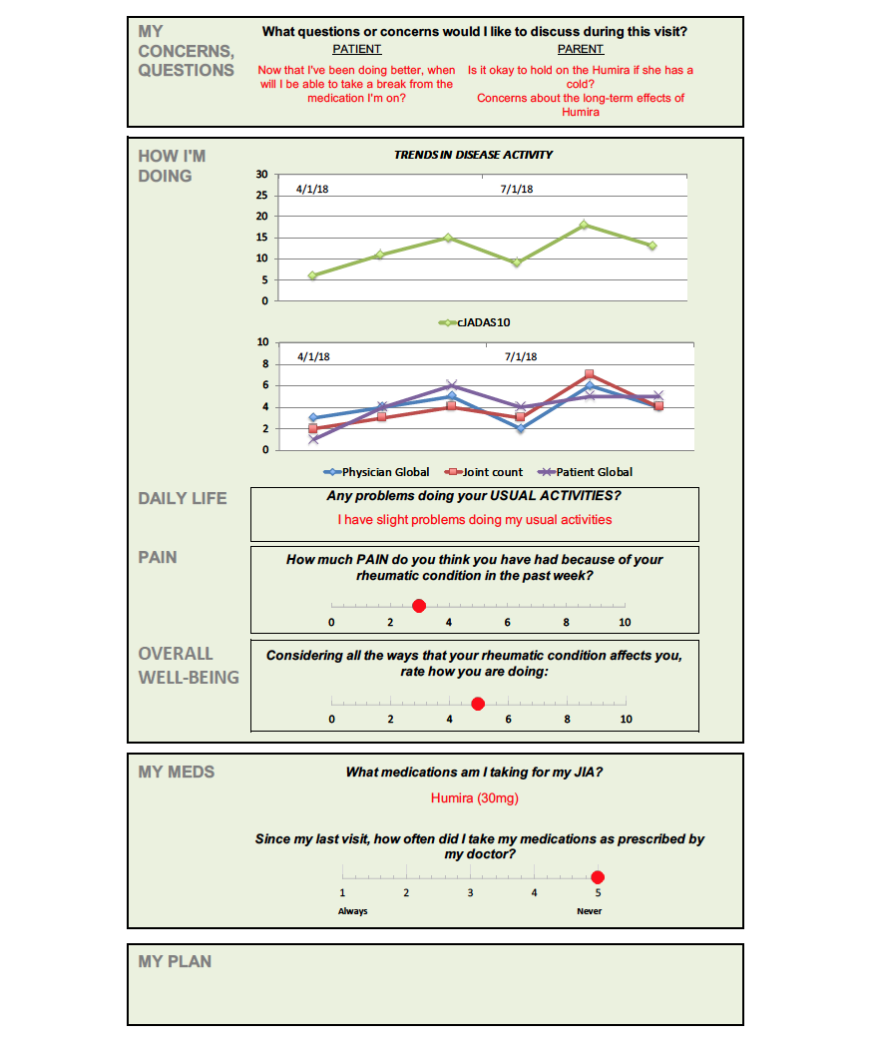
Paper-based dashboard prototype. cJADAS10: 10-joint clinical juvenile arthritis disease activity score.

**Figure 5:**
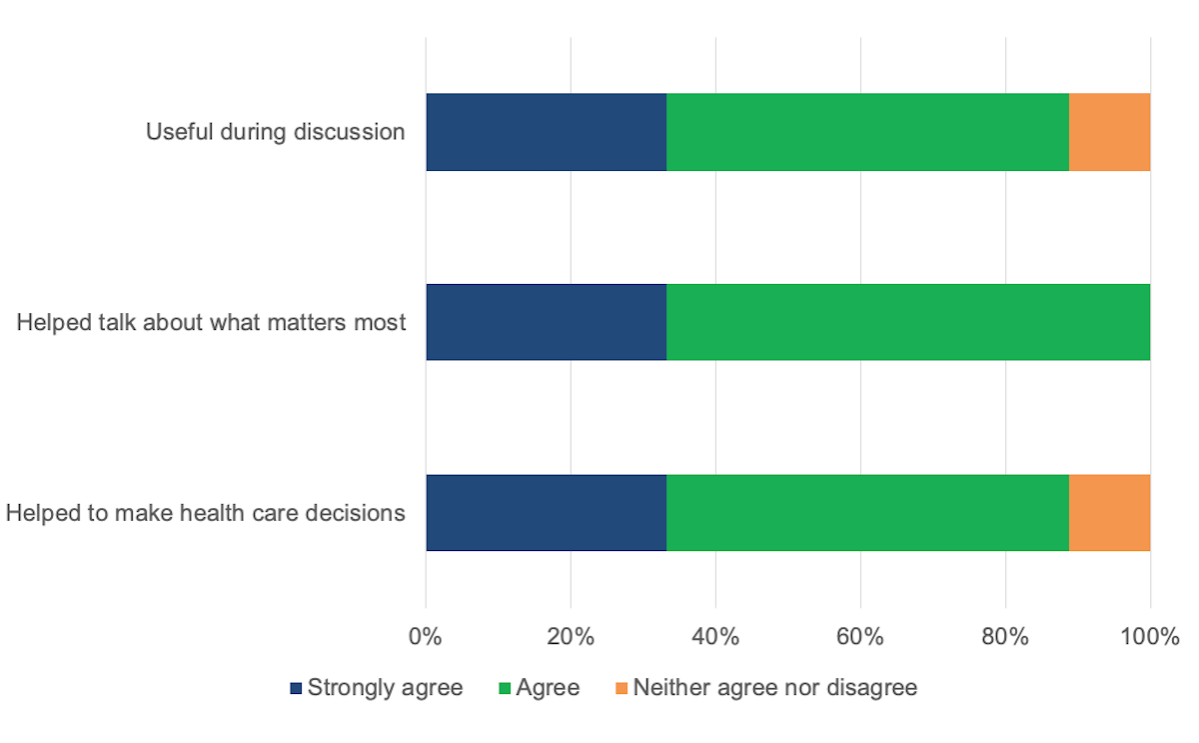
Dashboard low-tech testing: parent feedback (n=9).

**Figure 6:**
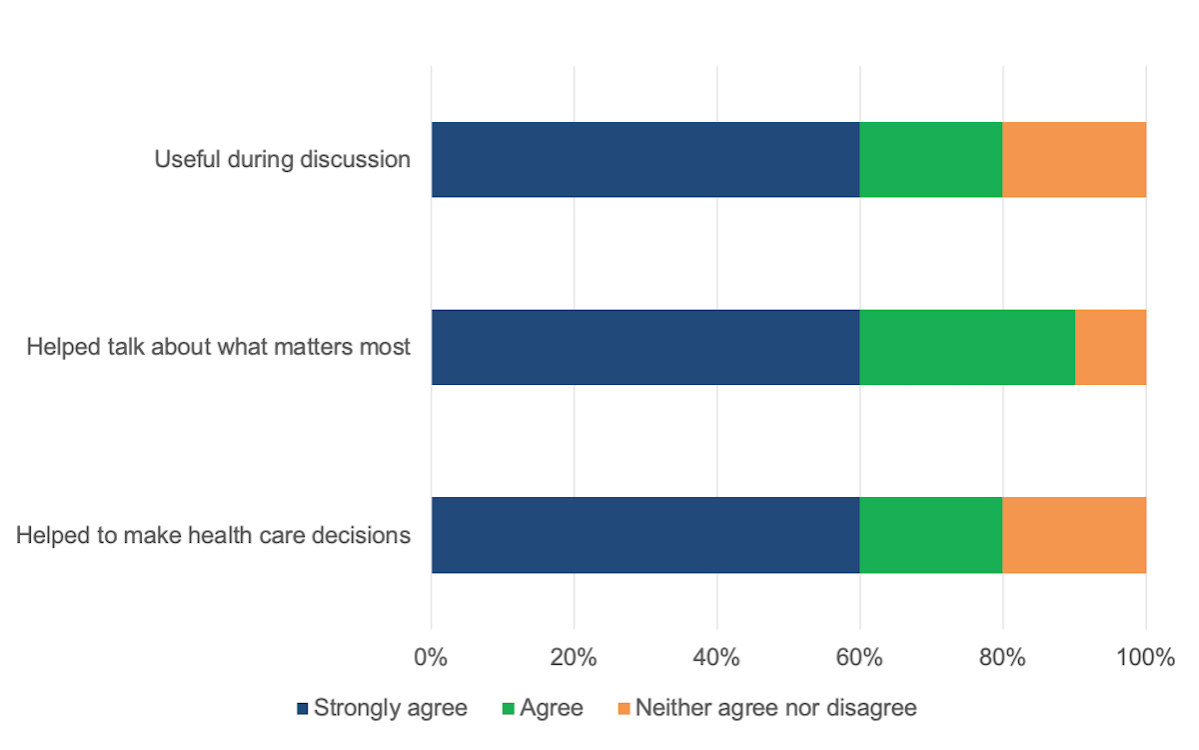
Dashboard low-tech testing: clinician feedback (n=10).

The paper-based prototype (Figure 4) was tested with 36 patients with JIA (aged 3-20 years; 24/36, 67% female) during a clinical visit. Parent (n=9) and clinician (n=10) feedback was very positive (Figures 5 and 6), with 89% (8/9) to 100% (9/9) of parents and 80% (8/10) to 90% (9/10) of clinicians strongly agreeing or agreeing that the dashboard (1) was useful during clinical discussions, (2) helped to talk about what mattered most, and (3) helped to make health care decisions.

Low-tech testing also uncovered important insights about the value of the dashboard, including the ability of patients and parents to share questions and concerns in advance of the visit, greater transparency in clinical assessment data routinely collected by clinicians and care teams (eg, joint count, physician global, and disease activity score), and the visualization of data over time to help make decisions. Although many PRO measures are still being validated for clinical use [30], patients and families are interested in seeing their scores in real time [31] and comparing them with past visits. The dashboard designs were iterated to include these elements and to improve the visual interface throughout the study.

Parents reported that being asked in advance about what they wanted to discuss most prevented them from forgetting anything during the visit. Clinicians appreciated understanding patient and parent concerns and ensured that these questions or concerns were addressed. One clinician shared an experience of how the responses highlighted the specific concerns of both the patient (*scared and did not want to restart medications*) and his parents (*concerned about setbacks from their child’s flare*), setting the framework for the visit. Another clinician reported, “We often forget to ask certain questions, and the dashboard *reminds* us to focus on the patient’s concerns rather than just looking at a clinical picture.”

Feedback on the usability of the paper-based prototype also yielded considerations for the dashboard design and data display, including (1) ensuring that the dashboard is updated on a real-time basis to include the current visit’s clinical assessment data as an important element of visualizing progress over time and engaging in shared decision-making, (2) clearly labeling the graphs for ease of reading and interpretation given varying scales of the data elements, and (3) orientation of the flow vertically to facilitate review and discussion.

**Figure 7:**
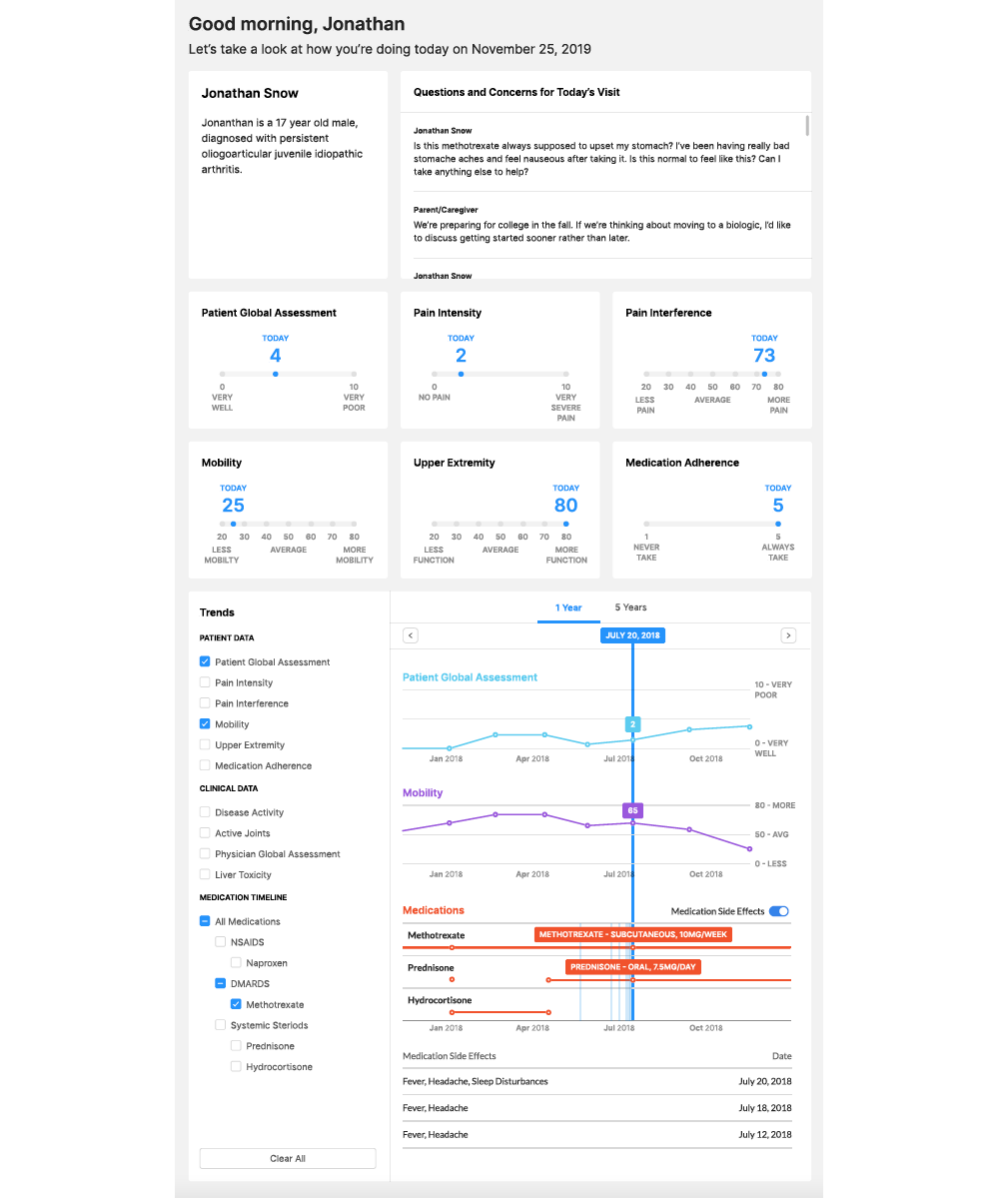
Final dashboard design wireframe.

The final dashboard design wireframe is illustrated in Figure 7. Three sections of the dashboard emerged: (1) patient and parent and family questions and concerns about the visit, (2) a snapshot of how the patient is currently doing, and (3) a trends section that longitudinally displays PROs, clinical data, and medications and reported side effects. The layout is intended to correspond to the clinical workflow. The top two sections provide the clinician with initial insight into the patient and family needs and how the patient is currently feeling, allowing for more rapid movement into the core of the visit. The trends section is intended for review after data from the clinical assessment have been captured and updated in real time.

## Discussion

### Principal Findings

This study used human-centered design principles to involve parents, patients, clinicians, and care team members in the development of a coproduction of care dashboards for clinical use in 4 diverse ambulatory pediatric rheumatology clinics across the United States. This process included evaluating the context of use and needs of end users, obtaining consensus on necessary data elements, and constructing a display prototype. Notable themes included the right data, in the right place, at the right time; data in once for multiple purposes; patient and family self-management components; and opportunity for education and increased transparency. A final set of 11 dashboard data elements was identified, including PROs, clinical data, and medications. Important design considerations include the incorporation of real-time data, clearly labeled graphs, and vertical orientation to facilitate reviews and discussions. Prototype paper-testing with 36 patients and families yielded positive feedback about the dashboard’s usefulness during clinic discussions, helped to talk about what mattered most, and informed health care decision-making.

Key components of the dashboard display included PROs, clinical data, and medications; all trended longitudinally with clearly labeled graphics and plans for real-time updates. Interestingly, a previsit agenda-setting question to be asked of both patients and families for “Questions or Concerns?” is deemed an important component of patient-centered care. This is consistent with findings in other coproduction projects in which patient and family questions and concerns helped to focus the content of the visits and prioritize what matters most to them [4,6]. In addition, our final dashboard prototype is similar to dashboards developed for adult RA [19] in their longitudinal presentation of PRO and clinical data along with medications, intended to enhance communication about how well medications work to improve patient symptoms, functioning, and disease activity.

Notably, all teams agreed that data collection should be streamlined and used for clinical care, collaborative improvement networks [13], and research, an important step toward the development of an integrated and sustainable learning health system [32]. Our prototype design encompasses the framework of having the right data at the right time to foster enhanced communication and collaboration during a clinical encounter and emphasizes the need for streamlined data collection to support multiple purposes and uses. Many of the dashboard data elements (functional status, pain score, patient global, joint count, and JADAS) were congruent with data collected within the CARRA [12] and PR-COIN [26] registries, as well as the Canadian JIA research network [33]. An opportunity exists to integrate these clinical care data with those required for research and quality improvement purposes. This integration represents a core pillar of building a learning health system [32] and allows for reduced burden on patients and families and more efficient data usage and optimization. Although our design is a paper prototype with an associated wireframe, the long-term goal is to develop an electronic dashboard integrated within the EHR. Notably, because all institutions use the same EHR, we anticipate that the use of standardized data mapping will facilitate interoperability among research networks.

Although prior work has been completed on dashboards in rheumatology [17,19], our study is unique from these published works in several notable ways. First, our study, which focused on the population with JIA, included 4 pediatric rheumatology teams, including patients and parents as team members, from diverse geographic locations who worked collaboratively throughout the co-design process. We also deployed a variety of human-centered design activities, including process mapping, personas, journey mapping, dashboard sketches, and observations. Third, our final set of dashboard data elements was greater than that of the adult population with RA [17,19]. Although both the adult and pediatric rheumatology dashboard data sets include a composite disease activity score, our teams felt that it was important to include component measures (physician global assessment, tender and swollen joint count, and patient global assessment) to promote greater understanding by patients and families regarding how this score is derived and used. Finally, and most notably, our set includes an agenda-setting question and questions regarding medication adherence and possible side effects to prompt discussion about medication intolerance, which can have a significant impact on quality of life [34].

### Strengths and Limitations

The strengths of our study include integrating patients and parents as full members of the pilot site teams, employing a variety of activities to gain insight into the needs of end users, and collecting qualitative and quantitative data to achieve consensus on a dashboard design. We used rapid-cycle iterative testing of a paper dashboard to simulate how a dashboard might work to support the coproduction cycle of (1) coassessing the patient’s current health status, (2) codeciding the next steps, (3) co-designing the care plan, and (4) codelivering care [35].

Although this study highlights the importance of involving end users in the design process, we acknowledge several potential limitations in our approach. We leveraged highly engaged clinical sites, care teams, and patients and families and used convenience sampling for development and testing; therefore, the perspectives we gained may not be representative of all pediatric rheumatology practices or populations. We engaged 4 diverse clinical sites, including small and large centers, in various locations across the United States. As we proceed to the next phase of building an electronic version of the dashboard, we will have the opportunity to test the dashboard and assess its usability and utility across a larger target population. Another limitation was the inability to pursue the design of a patient and family self-management tool. Patients and families expressed a strong desire to have a tool for individualized daily symptom tracking and note-taking to capture their experiences of living with a chronic disease. We acknowledge the importance of these functionalities [36]; however, the technological requirements for integrating them as part of a point-of-care dashboard were determined to be beyond the initial scope and capabilities of our study.

### Conclusions

We used a human-centered design process to actively engage patients with JIA, families, clinicians, and care teams to successfully create a blueprint for a point-of-care coproduction dashboard to foster meaningful conversations and shared decision-making about care and treatment plans. The necessary dashboard data elements include PROs, clinical data, and medications, and the display should use real-time data, have clearly labeled graphs, and a vertical orientation. Data capture that supports clinical care and research and improvement efforts is ideal. Results from dashboard testing indicated that it was useful during clinical discussions, helped to talk about what mattered most, and informed health care decision-making.

Future study efforts informed by this work and planned by the authors include (1) creating an electronic version of the point-of-care dashboard, (2) preparing for a successful launch through workflow integration and patient and family education efforts, (3) testing and implementing the dashboard at the 4 pediatric rheumatology pilot sites, and (4) conducting a formative evaluation of its usability and utility in supporting coproduction of care.

## Abbreviations

CARRA: Childhood Arthritis and Rheumatology Research Alliance
EHR: electronic health record
JADAS: Juvenile Arthritis Disease Activity Score
JIA: juvenile idiopathic arthritis
MoSCoW: must have, should have, could have, will not have
PR-COIN: Pediatric Rheumatology Care and Outcomes Improvement Network
PRO: patient-reported outcome
RA: rheumatoid arthritis
US: United States
